# The Relationship Between Sense of Community and Collaborative Learning: Quantitative Study in Medical Education

**DOI:** 10.2196/86893

**Published:** 2026-07-06

**Authors:** Harald Knof, Markus Berndt, Anne Herrmann-Werner, Bernhard Hirt, Thomas Shiozawa

**Affiliations:** 1 TIME – Tuebingen Institute for Medical Education, Faculty of Medicine, University of Tuebingen, Tuebingen, Germany; 2 Institute of Clinical Anatomy and Cell Analysis, Faculty of Medicine, University of Tuebingen, Elfriede-Aulhorn-Straße 8, Tuebingen, 72076, Germany, 49 7171 29 ext 73018; 3 Institute of Medical Education, LMU University Hospital, LMU Munich, Munich, Germany

**Keywords:** medical education, students, sense of community, collaborative learning, interpersonal relations, social identification, social environment, survey, regression analysis, factor analysis

## Abstract

**Background:**

Medical education has shifted from an individual, teacher-led process to an interactive, group-oriented approach, fostering clinical reasoning and teamwork. Sense of Community (SoC) appears to be a key factor in this process; however, its link to collaborative learning and academic success is underexplored.

**Objective:**

This study investigates this relationship, focusing on SoC dimensions (*connectedness* and *learning*) as well as the different forms of *initiative collaborative learning* and *subordinate collaborative learning*.

**Methods:**

The German form of the Classroom Community Scale (CCS-D) was used to assess SoC. The extent of collaborative learning was measured using the Learning Strategies in Study questionnaire. A total of 331 first-year medical students participated. Data analysis included exploratory factor analysis, correlation analysis, and regression analysis.

**Results:**

SoC showed a moderate positive correlation with the use of collaborative learning strategies (*r*=0.466; *P*<.001). *Connectedness* emerged as a significant predictor of collaborative learning (*R*²=0.257, adjusted *R*²=0.255; *F*_1,329_=113.990; *P*<.001). Both *initiative collaborative learning* and *subordinate collaborative learning* are based more strongly on feelings of *connectedness*. The feeling that members of the course depend on oneself had the strongest predictive value for collaborative learning (*R*²=0.188, adjusted *R*²=0.185; *F*_1,329_=75.956; *P*<.001).

**Conclusions:**

A strong SoC promotes the use of collaborative learning strategies, particularly through social *connectedness*. The lesser importance of the *learning* dimension suggests that social bonds appear more influential than shared academic goals, particularly during help seeking, where trust and psychological safety are critical. Targeted support of learning communities may enhance didactic approaches and foster both *initiative collaborative learning* and *subordinate collaborative learning*. Cultivating connectedness and responsibility may support academic adaptation and emotional well-being in the critical early phase of medical education.

## Introduction

### Background

Learning has shifted from an individual and teacher-directed process to a more interactive, group-oriented approach [1]. Knowledge is therefore constructed collaboratively to enhance knowledge acquisition, clinical reasoning, and teamwork skills [2-4]. The theoretical roots can be traced back to Piagetian and Vygotskian perspectives, both emphasizing the role of social interactions in learning [5,6]. In the Piagetian theory, sociocognitive conflicts drive learning by exposing individuals to alternative problem-solving strategies [5,7-10]. These conflicts encourage cognitive restructuring, deeper understanding, and skill development [11,12]. The Vygotskian perspective highlights the so-called *zone of proximal development*, where learning occurs through guided social interaction and peer support until learners achieve independence [6,7,10,13].

Contemporary educational research further highlights the importance of leadership, learning culture, and socially supportive educational environments in shaping successful learning processes [14-18]. Within modern “leadership for learning” approaches, educators are increasingly understood not merely as instructors but as mediators who create psychologically safe, collaborative, and student-centered learning environments [14-17]. More recent perspectives, such as socially shared regulation of learning, conceptualize learning as a process in which learners manage motivation, cognition, and learning activities through interaction, feedback, and shared responsibility within groups [19-21]. Similarly, the communities of practice theory emphasizes that learning and professional development occur through active participation in socially and professionally meaningful communities [22,23].

These developments are also reflected in competency-based medical education frameworks such as entrustable professional activities, which underline the acquisition of professional responsibility, self-regulated learning, feedback, and collaborative practice within authentic clinical contexts [24]. Together, these modern perspectives increasingly conceptualize learning not only as knowledge acquisition but as a socially embedded, collaborative, and professionally oriented developmental process.

### Collaborative Learning

Collaborative learning is a widely studied but inconsistently defined concept, often used interchangeably with terms such as *group work* or *tutorial learning* [25]. It refers to situations where 2 or more individuals learn together by joint problem-solving and pursuing shared goals [26,27]. Two forms can be distinguished: *initiative collaborative learning*, which is proactive, and *subordinate collaborative learning*, which is reactive and occurs when students seek help after encountering difficulties [28]. Collaborative learning offers numerous advantages across multiple layers and levels [29]. Cognitively, it promotes argumentation, questioning, and explanation, deepening understanding for both the explainer and the recipient [30-32]. Culturally, it fosters a learning environment that values professional exchange [33]. It encourages the identification of knowledge gaps, explanation, questioning, and clarification, leading to deeper learning [34-37]. Professionally, it supports communication and teamwork skills essential in fields such as medicine, where complex knowledge must be conveyed clearly and collaboratively [38,39].

In addition, collaborative learning influences cognitive, emotional, and motivational regulation, as conceptualized in socially shared regulation of learning [19-21]. Learners engage in self-shared, coshared, and socially shared regulation to refine strategies and sustain engagement [19,21,40]. These shared responsibilities and mutual support create a Sense of Community (SoC), which plays a key role in sustaining motivation and fostering a positive learning environment [1,41].

### Sense of Community

Despite extensive research, there is no universally accepted definition of the term “Sense of Community” [42-45]. Some definitions emphasize belonging and emotional connection, while others focus on interdependence and shared identity [46,47]. The study by McMillan and Chavis [44] describes SoC as a feeling of belonging, mutual significance, and confidence that members’ needs will be met through group commitment [45]. Core elements include trust, responsibility, shared goals, and mutual interdependence [42,48-51]. The expression of SoC, however, varies by context, particularly in educational settings [48,52,53].

In classrooms, community takes a distinct form shaped by learning goals, structured interactions, and time-limited membership [54]. It consists of 2 main components: *connectedness*, reflecting belonging and trust, and *learning*, representing shared academic goals [42,55]. Learners who experience a high sense of classroom community are better able to coordinate working together to achieve the group’s collective goals [56]. A strong classroom community promotes engagement, collaboration, and resilience [57,58]. Weak community ties instead increase the risk of disengagement and dropout [59].

Belonging appears to be a fundamental psychological need that directly influences motivation, engagement, and persistence in educational contexts [60,61]. This perspective is further extended by the concept of communities of practice, which frames learning as participation in a shared professional culture and the gradual development of identity through social interaction [22,23]. Educators therefore play a crucial role in fostering connectedness and collective learning to enhance student satisfaction and success [62,63].

Both SoC and collaborative learning contribute to learning and may lead to academic success. These concepts share commonalities, may be linked, or build a communal foundation for social learning. Till date, there is no research connecting these 2 concepts or investigating both constructs in the same study population. This study aims to unveil the interrelations between SoC and collaborative learning based on 2 validated questionnaires [64-66]. The research questions (RQs) can be formulated as follows:

- RQ1: What is the relationship between SoC and collaborative learning?
- RQ2: How do connectedness and learning aspects influence collaborative learning?
- RQ3: How does the SoC differentially influence *initiative collaborative learning* and *subordinate collaborative learning*?
- RQ4: Which items of the German form of the Classroom Community Scale (CCS-D) are key predictors of collaborative learning?

## Methods

### Data Collection

First-semester medical students from the winter semester 2021-2022 and summer semester 2022 cohorts were invited to participate in a paper-based survey exploring their learning strategies and perceived SoC. By this time, participants had completed all courses for the first semester of their medical studies. The initial invitation to participate was extended personally by the first author. Students received a €5 (€1=US $1.15 as of June 8, 2026) expense allowance for their participation.

### Ethical Considerations

Prior to the study, written informed consent was obtained from all participants, who were thoroughly informed about the study’s opportunities, risks, rights, obligations, and voluntary nature. Data collection was conducted in a pseudonymized format, with all participants consenting to anonymized data publication. Students retained the right to withdraw consent at any time without facing any disadvantages. Ethics approval for the study was granted by the ethics committee at Eberhard Karls University of Tuebingen (reference number 086/2022BO2). The study was carried out in accordance with the Declaration of Helsinki.

### Measures

#### German Version of the Classroom Community Scale

To assess the perceived SoC, the CCS-D was used [64] (Table 1). On the basis of Rovai’s original Classroom Community Scale (CCS), this version has been validated for use in German-speaking contexts [64,67].

**Table 1.** German form of the Classroom Community Scale.

Item	Subscale	Prompt
1	Connectedness	I feel that students in this course care about each other.
2	Learning	I feel that I am encouraged to ask questions.
3	Connectedness	I feel connected to others in this course.
4i	Learning	I feel that it is hard to get help when I have a question.
5i	Connectedness	I do not feel a spirit of community.
6	Learning	I feel that I receive timely feedback.
7	Connectedness	I feel that this course is like a family.
8i	Learning	I feel uneasy exposing gaps in my understanding.
9i	Connectedness	I feel isolated in this course.
10i	Learning	I feel reluctant to speak openly.
11	Connectedness	I trust others in this course.
12i	Learning	I feel that this course results in only modest learning.
13	Connectedness	I feel that I can rely on others in this course.
14i	Learning	I feel that other students do not help me learn.
15	Connectedness	I feel that members of this course depend on me.
16	Learning	I feel that I am given ample opportunities to learn.
17i	Connectedness	I feel uncertain about others in this course.
18i	Learning	I feel that my educational needs are not being met.
19	Connectedness	I feel confident that others will support me.
20i	Learning	I feel that this course does not promote a desire to learn.

The CCS consists of 20 items divided into 2 subscales: *connectedness* and *learning*. The *connectedness* subscale focuses on feelings of connection, while the *learning* subscale addresses perceptions of interaction within the learning community. Participants rate each item on a 5-point Likert scale. The scoring for items 1, 2, 3, 6, 7, 11, 13, 15, 16, and 19 is as follows: *strongly agree=4, agree=3; neutral=2, disagree=1, and strongly disagree=0*. For items 4, 5, 8, 9, 10, 12, 14, 17, 18, and 20, the scoring scale is reversed: *strongly agree=0, agree=1, neutral=2, disagree=3, and strongly disagree=4* [67]. For further analysis, these item scores must be inverted. These items are labeled with an “i” in the tables and figures of this paper.

To calculate the total CCS score (maximum score=80), the values of all 20 items are summed. Each subscale has a maximum score of 40. Higher scores on the total CCS score reflect a stronger sense of classroom community, while lower scores indicate a weaker sense of classroom community [67].

#### Learning Strategies in Study Questionnaire

To assess the use of collaborative learning strategies, the Learning Strategies in Study (LIST) questionnaire found in the study by Wild et al [65,66] was used (Table 2). In this survey, only the *learning with fellow students* subscale of the LIST questionnaire was used. Items 1 to 4 form the subscale for *initiative collaborative learning*, while items 5 to 7 form the subscale for *subordinate collaborative learning* [28]. Participants rate the frequency of their use of each learning strategy on a 5-point Likert scale (*1=very seldom*, *2=seldom*, *3=sometimes*, *4=often*, and *5=very often*) [65]. The extent of the use of collaborative learning strategies was determined by calculating the scale mean, with higher values indicating greater use.

**Table 2.** Learning Strategies in Study scale—learning with fellow students.

Item	Prompt
1	I work on texts or assignments together with my fellow students.
2	I take time to discuss the material with fellow students.
3	I compare my lecture notes with those of my fellow students.
4	I have a fellow student quiz me and ask him questions about the material as well.
5	I enlist the help of others when I have serious comprehension problems.
6	If something is not clear to me, I ask a fellow student for advice.
7	If I discover major gaps in my records, I contact my fellow students.

### Data Analysis

Following a descriptive analysis, Pearson correlation coefficients were calculated, and linear regression analyses were conducted. Subsequently, a principal component analysis (PCA) with varimax rotation and Kaiser normalization was conducted to verify the underlying structure and confirm the division into *subordinate collaborative learning* and *initiative collaborative learning* of the *learning with fellow students* scale. The PCA used listwise deletion, and items with factor loadings ≤ |0.30| on one or both dimensions were retained [68,69]. Prior to the PCA, the Kaiser-Meyer-Olkin measure of sampling adequacy and Bartlett test of sphericity were assessed. The PCA results served as the foundation for subsequent stepwise regression analyses. The significance level for evaluating results was set at .05.

SPSS Statistics (version 29; IBM Corp) was used for collecting questionnaire responses, data extraction, and statistical analysis.

## Results

### Sample

A total of 344 first-semester medical students were surveyed. With a total number of 210 students per semester, a maximum of 420 participants could have been included, resulting in a response rate of 81.9% (344/420). Participants with missing data were excluded, resulting in a final sample of 331 respondents. The study sample reflected the gender distribution of the cohort, with 68.9% (n=228) of participants being female, and the ages of the participants ranged from 18 to 35 years, with a mean age of 20.72 (SD 2.72) years. The majority of participants were medical students (320/331, 96.7%), followed by dentistry students (10/331, 3%) and students from molecular medicine (1/331, 0.3%).

### Scale Characteristics and Internal Consistency

Descriptive statistics for the individual items, as well as the total CCS-D score and its subscale scores, are presented in Table S1 in Multimedia Appendix 1. The total CCS-D demonstrated good internal consistency (Cronbach α=0.87) as a measure for reliability. The *connectedness* subscale (Cronbach α=0.84) also showed good internal consistency, while the *learning* subscale (Cronbach α=0.77) showed acceptable internal consistency. Similarly, Table S2 in Multimedia Appendix 1) presents descriptive statistics of the LIST questionnaire along with the total score on the *learning with fellow students* scale and its subscales. The total *learning with fellow students* scale showed good internal consistency (Cronbach α=0.86). The subscales for *initiative collaborative learning* (Cronbach α=0.82) and *subordinate collaborative learning* (Cronbach α=0.84) demonstrated high reliability.

### RQ1: Relationship Between SoC and Collaborative Learning

**Figure 1.**
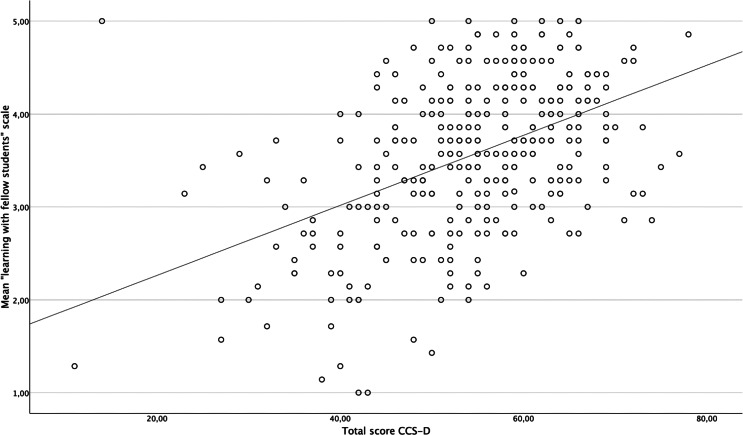
Scatterplot of the total score of the German form of the Classroom Community Scale (CCS-D) and the *learning with fellow students* subscale of the Learning Strategies in Study questionnaire (LIST). Each point represents an individual student (N=331).

To measure the relationship between SoC and collaborative learning, Pearson correlation was conducted. The correlation coefficient for the relationship between the SoC and the *learning with fellow students* scale was found to be *r*=0.466; *P*<.001. Higher levels of SoC were associated with greater use of collaborative learning strategies (Figure 1).

To further examine this relationship, the next step was to examine the association between SoC and collaborative learning through linear regression analysis. The regression model explained 21.7% of the variance in the use of collaborative learning strategies (*R*²=0.217, adjusted *R*²=0.214; *F*_1,329_=91.020; *P*<.001). The unstandardized coefficient was B=0.038 and the standardized coefficient was ß=0.466, both statistically significant at *P*<.001. SoC was significantly associated with the use of collaborative learning strategies.

### RQ2: Influence of *Connectedness* and *Learning* on Collaborative Learning

To determine whether *connectedness* or *learning* is more strongly associated with collaborative learning, a stepwise regression analysis was conducted using CCS-D subscales *connectedness* and *learning* as predictors, with the *learning with fellow students* scale as the dependent variable (Table S3 in Multimedia Appendix 1).

The model including only *connectedness* yielded significant results: *R*^2^=0.257, adjusted *R*^2^=0.255; *F*_1,329_=113.990; *P*<.001. The unstandardized coefficient B=0.070 and the standardized coefficient ß=0.507 were both significant at *P*<.001. The *learning* subscale was excluded from the model (β=0.023; 2-tailed *t*_329_=0.382; *r*=0.021, *T*=0.651; *P*=.70).

### RQ3: Differences in the Influence of SoC on Initiative Collaborative Learning and Subordinate Collaborative Learning

#### Principal Component Analysis

To verify the division between *initiative collaborative learning* and *subordinate collaborative learning*, a PCA was conducted. The Kaiser-Meyer-Olkin measure was 0.844, representing a relatively good factor analysis. The Bartlett test of sphericity was significant (approximately *χ^2^*=1120.7; *P*<.001), suggesting that the analyzed data do not result in an identity matrix and are therefore suitable for factor analysis. Only factors with eigenvalues ≥1 were considered [70,71]. On the basis of the Kaiser criteria and the scree plot, 2 factors with eigenvalues ≥1 were retained, accounting for 70.95% of the total variance. In the varimax-rotated 2-factor model (Table 3), the underlying structure of *subordinate collaborative learning* and *initiative collaborative learning* can be verified, as the items 1 to 4 are loading on factor 1 and the items 5 to 7 on factor 2 [28].

**Table 3.** Principal component analysis of *learning with fellow students*^a^^,^^b^.

Rotated component matrix^c^	Component	
	1	2
Item 1	0.840	—^d^
Item 2	0.838	—
Item 3	0.641	—
Item 4	0.776	—
Item 5	—	0.854
Item 6	—	0.852
Item 7	—	0.808

a Extraction method: principal component analysis. Rotation method: varimax with Kaiser normalization.

b Factor loadings <.35 are not reported.

c Rotation converged in 3 iterations.

d Not applicable.

#### Stepwise Regression

To investigate which factor was more strongly related to *initiative collaborative learning* and *subordinate collaborative learning* strategies, stepwise regression analyses were conducted using the CCS-D subscales *connectedness* and *learning* as predictors, with the subscales *initiative collaborative learning* and *subordinate collaborative learning* as outcome variables.

For *initiative collaborative learning*, the model including only *connectedness* yielded significant results, with *R*²=0.157, adjusted *R*²=0.155, *F*_1,329_=61.419; *P*<.001 (Table S4 in Multimedia Appendix 1). The unstandardized coefficient was B=0.065 and the standardized coefficient was ß=0.397, both statistically significant at *P*<.001. The *learning* subscale was not retained from the model (β=–0.013; *t*_329_=−0.208; *r*=−0.012; *T*=0.651; *P*=.83).

For *subordinate collaborative learning*, the model including *connectedness* also showed significant results, with *R*²=0.277, adjusted *R*²=0.275; *F*_1,329_=125.976; *P*<.001 (Table S5 in Multimedia Appendix 1). The unstandardized coefficient was B=0.077 and the standardized coefficient was ß=0.526, both statistically significant at *P*<.001. The *learning* subscale was again not retained from the model (β=0.071; *t*_329_=1.214; *r*=0.067; *T*=0.651; *P*=.22).

### RQ4: CCS-D Items Associated With Collaborative Learning

To determine which items are most strongly associated with collaborative learning, a stepwise regression analysis was conducted using the CCS-D items as independent variables, with the scale *learning with fellow students* as the dependent variable.

The final model, which included item 15, item 3, item 14i, item 9i, and item 19, yielded significant results: *R*²=0.338, adjusted *R*²=0.328; *F*_5,325_=33.145; *P*<.001. The unstandardized coefficients were B=0.211 (*P*<.001) for item 15, B=0.130 (*P*=.007) for item 3, B=0.131 (*P*=.002) for item 14i, B=0.128 (*P*=.009) for item 9i, and B=0.138 (*P*=.01) for item 19. The standardized coefficients ß ranged from ß=0.139 to ß=0.244, indicating that item 15 (“I feel that members of this course depend on me”) showed the strongest association with collaborative learning (ß=0.244), followed by item 14i (ß=0.156) and item 19 (ß=0.139; Table S6 in Multimedia Appendix 1).

## Discussion

### Principal Findings

This study aimed to explore the relationship between SoC and collaborative learning among first-semester medical students. The results of this study show that perceived SoC is significantly associated with the use of collaborative learning strategies. The underlying structure of *subordinate collaborative learning* and *initiative collaborative learning* was confirmed. *Connectedness* showed a stronger association with collaborative learning than the *learning* dimension. *Connectedness* was more strongly associated with *subordinate collaborative learning* than with *initiative collaborative learning*. Item 15 of the CCS-D (“I feel that the members of this course depend on me”) showed the strongest association with collaborative learning.

### RQ1: Relationship Between SoC and Collaborative Learning

The findings resonate with theoretical perspectives that link social and emotional bonds to cooperative learning [5,6]. A stronger SoC is associated with a greater engagement in cooperative learning, supporting prior research highlighting the importance of social belonging in promoting student engagement [42,45].

This is particularly relevant for first-semester medical students who must navigate both academic and social transitions [72]. Up to this point of their studies, many students have little experience with self-organized group learning and must first familiarize themselves with new learning demands and environments [73]. School-based learning strategies must be adapted to the new university requirements at the beginning of studies [74]. In this transitional phase, social connections can help by reducing uncertainties and lowering the threshold for interaction [75]. Numerous studies show that both learning environment and interaction with fellow students play a central role in the selection and adaptation of learning strategies [76-79]. Collaborative learning helps reduce exam anxiety and related stress, which are known to hinder academic success [80,81]. Students who feel well-prepared are more likely to remain motivated and persist through academic challenges [82,83].

### RQ2: Influence of *Connectedness* and *Learning* on Collaborative Learning

In this study, only *connectedness* was significantly associated with collaborative learning, while *learning* was not retained from the analytical models. This suggests that interpersonal connectedness is more strongly associated with collaborative learning behavior than sharing common academic goals. One possible explanation for this lies in the specific structure of medical studies, which is more characterized by external learning objectives (eg, exams and clinical competencies). These standardized and homogeneous goals may be less relevant for collaborative behavior. Studies further show that SoC serves as a mediator between motivation and student contribution during collaborative learning, with the mediation effect being stronger for intrinsic motivation than for extrinsic motivation [84]. Therefore, interpersonal connectedness not only appears to facilitate social integration but also strengthens intrinsic motivation, which in turn significantly contributes to active participation in group learning [84]. The first term of medical school is a phase in which students need to orient themselves both academically and socially as written above. In this orientation phase, feelings of belonging and trust are often more relevant than clearly defined common learning goals. It is therefore plausible that interactions in early study phases primarily serve social exchange and network building, while learning goals gain secondary importance.

Another interpretation is that social distancing during the COVID-19 pandemic may have increased the importance of interpersonal connection over content motivation in developing collaborative learning. During the pandemic, opportunities to build personal relationships and engage in face-to-face interactions were severely restricted, as universities and schools shifted to digital learning environments [85-89]. The participants in this study had to complete high school during that time under remote learning conditions [90]. Many students reported missing in-person interaction and expressed a need to meet and learn together to stay motivated [91,92]. These prior experiences may have intensified the value of social connectedness as a foundational support in their early university learning. In this context, feeling connected to peers may serve not only as a motivator for collaborative learning but also as a compensatory mechanism for previously unmet social and educational needs.

### RQ3: Differences in the Influence of SoC on Initiative Collaborative Learning and Subordinate Collaborative Learning

Interestingly, *connectedness* is more strongly associated with *subordinate collaborative learning* than *initiative collaborative learning*. This difference can be understood by considering their distinct natures. *Initiative collaborative learning* is proactive when students actively engage with peers to work through tasks, discuss lecture content, and question one another to strengthen understanding [28]. In contrast, *subordinate collaborative learning* is reactive, arising when students encounter challenges such as unclear concepts or incomplete notes and turn to peers for support to fill those gaps and improve comprehension [28]. This differentiation and our findings suggest that social factors are particularly relevant when students rely on support, whereas initiative is more likely driven by individual traits such as motivation or self-efficacy [93-95].

In highly selective programs such as medicine in Germany, where all students have been admitted based on outstanding academic achievement, asking for help is not only a question of competence but also social safety [96]. When all peers may be considered intellectually capable, students may hesitate to seek help from someone if they fear being judged or feeling help seeking as evidence of incompetence [96-99]. In this context, interpersonal trust becomes a crucial factor for starting collaborative behaviors [100]. Particularly in *subordinate collaborative learning*, students are maybe more likely to turn to peers who create a nonthreatening environment and signal mutual respect. This supports the finding that social connectedness, more than academic motivations and shared learning goals, is associated with collaborative learning strategies. Trust creates a psychologically safe space in which students can take academic risks without fear of judgment [101]. Feeling embedded in a supportive peer community may lower the psychological threshold for admitting uncertainty and promotes help seeking without fear of negative evaluation [100,101].

### RQ4: CCS-D Items Associated With Collaborative Learning

Particularly noteworthy is the role of item 15 of the CCS-D (“I feel that the members of this course depend on me”), which showed the strongest association with collaborative learning. This highlights the importance of perceived responsibility within a learning community and aligns with the concept of a “Sense of Community Responsibility” in collaborative environments [50,51].

Accordingly, psychological experiences of responsibility and commitment among members of a community are significantly linked to their attitudes and behaviors within collaborative processes [102]. It has also been demonstrated that collaborative learning fosters a sense of accountability, as students feel responsible not only for their own performance but also for that of their peers [103]. Research has shown that individuals tend to emphasize group cohesion when the value of the group is threatened, with such reactions being most pronounced by those members who already feel a strong sense of commitment toward other group members [102,104]. Feeling that others depend on one’s contribution may strengthen identification with the group, which in turn increases the motivation to participate actively and contribute meaningfully. This aligns with social identity theory, which posits that individuals derive part of their self-concept from group membership [105]. Within learning contexts, a sense of connectedness and perceived responsibility toward peers can foster intrinsic motivation and engagement, thereby promoting collaborative behaviors and support [106].

It is also worth discussing whether the present finding may, in part, reflect a form of “helper syndrome,” in which individuals derive a sense of self-worth from being needed by others and perceive their peers as dependent on their contributions [107]. In the context of health care professionals and medical education, where altruism, care, and accountability are core professional values, this perceived responsibility may be reinforced by internalized norms of helping and supporting others. The statement “they depend on me” likely captures a positive perception of having a meaningful role within the group, which can be intrinsically motivating for collaborative learning as individuals experience social worth when they feel needed [108,109]. While such attitudes can strengthen engagement and cooperative behaviors, they may also be associated with a tendency to prioritize others’ needs over one’s own well-being, or to underestimate personal limits, which are characteristic aspects of helper syndrome [110].

### Strengths and Limitations

This study has several strengths. Data were collected across multiple cohorts, providing a broader and more generalizable view of first-semester medical students. The high response rate reduces selection bias, and the use of validated instruments strengthens construct validity for both SoC and collaborative learning measures. Robust statistical analyses, including principal component and regression analyses, further enhance methodological rigor.

However, some limitations should be noted. The LIST questionnaire may not fully capture the range of collaborative learning strategies actually used, and self-reported data are susceptible to recall and response bias. Furthermore, all students participated in fixed-group laboratory courses and mentoring programs that fostered collaboration and community, which may have elevated CCS-D scores. Still, as all participants experienced the same conditions, a consistent baseline across the sample can be assumed. A moderate proportion of variance in collaborative learning behavior was explained by the models, while a substantial proportion remained unexplained. This may be attributed to the use of stepwise regression analyses, which are prone to chance findings, thereby potentially compromising the stability and generalizability of the identified predictors. Similarly, the cross-sectional design does not allow for causal inferences, meaning that the observed relationships can only be interpreted as associations. The results should therefore be interpreted with appropriate caution.

### Conclusions

A strong SoC appears to be closely associated with collaborative learning in medical education. Social *connectedness* showed a stronger association with collaborative learning than shared academic goals. Both *initiative collaborative learning* and *subordinate collaborative learning* benefited from stronger community ties, with the effect being most pronounced for reactive peer support. The feeling that course members depend on one’s own contributions showed the strongest association for collaborative learning.

These findings suggest that deliberately cultivating SoC through structured group work, peer networks, and mentoring may support not only learning outcomes but also teamwork skills and collaboration. Beyond the academic context, fostering a strong sense of belonging and mutual support may better prepare future health care professionals for the collaborative demands of clinical practice, where trust, communication, and cooperation are essential.

## Supplementary material

10.2196/86893 Multimedia Appendix 1 Descriptive statistics and stepwise regression analyses.

## Abbreviations

CCS: Classroom Community Scale
CCS-D: German form of the Classroom Community Scale
LIST: Learning Strategies in Study
PCA: principal component analysis
RQ: research question
SoC: Sense of Community
