# Supplementary material for: The Relationship Between Sense of Community and Collaborative Learning: Quantitative Study in Medical Education
Source: JMIR Med Educ. 2026 Jul 6;12:e86893. doi: 10.2196/86893 (PMC13334800; doi:10.2196/86893)
Supplement: Multimedia Appendix 1 [file mededu-v12-e86893-s001.docx]

## Multimedia Appendix 2

**Table A:**

**Descriptive statistics of individual items and subscales of CCS-D**

| *Descriptive Statistics of individual items and subscales of CCS-D* | | | | | |
| --- | --- | --- | --- | --- | --- |
|  | N | Minimum | Maximum | Mean | Std. Deviation |
| Item 1 | 331 | 0 | 4 | 3.13 | .802 |
| Item 2 | 331 | 0 | 4 | 2.56 | .903 |
| Item 3 | 331 | 0 | 4 | 2.88 | .993 |
| Item 4 i | 331 | 0 | 4 | 2.95 | .963 |
| Item 5 i | 331 | 0 | 4 | 3.14 | 1.023 |
| Item 6 | 331 | 0 | 4 | 2.35 | .940 |
| Item 7 | 331 | 0 | 4 | 1.54 | 1.088 |
| Item 8 i | 331 | 0 | 4 | 2.40 | 1.017 |
| Item 9 i | 331 | 0 | 4 | 3.19 | .915 |
| Item 10 i | 331 | 0 | 4 | 2.86 | 1.013 |
| Item 11 | 331 | 0 | 4 | 2.80 | .804 |
| Item 12 i | 331 | 0 | 4 | 2.85 | .890 |
| Item 13 | 331 | 0 | 4 | 2.79 | .869 |
| Item 14 i | 331 | 0 | 4 | 2.95 | 1.000 |
| Item 15 | 331 | 0 | 4 | 2.31 | .968 |
| Item 16 | 331 | 0 | 4 | 2.67 | 1.077 |
| Item 17 i | 331 | 0 | 4 | 2.30 | 1.008 |
| Item 18 i | 331 | 0 | 4 | 2.87 | 1.001 |
| Item 19 | 331 | 0 | 4 | 2.88 | .844 |
| Item 20 i | 331 | 0 | 4 | 2.88 | .951 |
| **CCS-D score Total** | **331** | **11.00** | **78.00** | **54.3202** | **10.36194** |
| CCS-D score *Connectedness* | 331 | 3.00 | 40.00 | 26.9517 | 6.07808 |
| CCS-D score *Learning* | 331 | 7.00 | 40.00 | 27.3686 | 5.53748 |
| Valid N (listwise) | 331 |  |  |  |  |

**Table B:**

**Descriptive Statistics of individual items and subscales of LIST**

| *Descriptive Statistics of individual items and subscales of LIST* | | | | | |
| --- | --- | --- | --- | --- | --- |
|  | N | Minimum | Maximum | Mean | Std. Deviation |
| Item 1 | 331 | 1 | 5 | 3.21 | 1.234 |
| Item 2 | 331 | 1 | 5 | 3.60 | 1.140 |
| Item 3 | 331 | 1 | 5 | 2.48 | 1.296 |
| Item 4 | 331 | 1 | 5 | 3.53 | 1.253 |
| Item 5 | 331 | 1 | 5 | 4.02 | 1.037 |
| Item 6 | 331 | 1 | 5 | 4.14 | .939 |
| Item 7 | 330 | 1 | 5 | 3.91 | 1.078 |
| **LIST score Collaborative Learning total** | **331** | **1.00** | **5.00** | **3.5564** | **.83885** |
| LIST score Initiative Collaborative Learning | 331 | 1.00 | 5.00 | 3.2069 | .99499 |
| LIST score Subordinate Collaborative Learning | 331 | 1.00 | 5.00 | 4.0237 | .88736 |
| Valid N (listwise) | 330 |  |  |  |  |

**Table C:**

**Stepwise regression analysis: Influence of *Connectedness* and *Learning* on Collaborative Learning**

| *Model Summary* | | | | |
| --- | --- | --- | --- | --- |
| Model | R | R Square | Adjusted R Square | Std. Error of the Estimate |
| 1 | ,507^a^ | ,257 | ,255 | ,72401 |
| a. Predictors: (Constant), CCSD_Connectedness | | | | |

| *ANOVA^a^* | | | | | | |
| --- | --- | --- | --- | --- | --- | --- |
| Model | | Sum of Squares | df | Mean Square | F | Sig. |
| 1 | Regression | 59,753 | 1 | 59,753 | 113,990 | <,001^b^ |
|  | Residual | 172,459 | 329 | ,524 |  |  |
|  | Total | 232,211 | 330 |  |  |  |
| a. Dependent Variable: M_LIST_total_Collaboration | | | | | | |
| b. Predictors: (Constant), CCSD_Connectedness | | | | | | |

| *Coefficients^a^* | | | | | | |
| --- | --- | --- | --- | --- | --- | --- |
| Model | | Unstandardized Coefficients | | Standardized Coefficients | t | Sig. |
|  |  | B | Std. Error | Beta |  |  |
| 1 | (Constant) | 1,670 | ,181 |  | 9,216 | <,001 |
|  | CCSD_Connectedness | ,070 | ,007 | ,507 | 10,677 | <,001 |
| a. Dependent Variable: M_LIST_total_Collaboration | | | | | | |

| *Excluded Variables^a^* | | | | | | |
| --- | --- | --- | --- | --- | --- | --- |
| Model | | Beta In | t | Sig. | Partial Correlation | Collinearity Statistics |
|  |  |  |  |  |  | Tolerance |
| 1 | CCSD_Learning | ,023^b^ | ,382 | ,703 | ,021 | ,651 |
| a. Dependent Variable: M_LIST_total_Collaboration | | | | | | |
| b. Predictors in the Model: (Constant), CCSD_Connectedness | | | | | | |

**Table D:**

**Stepwise regression analysis: Influence of *Connectedness* and *Learning* on *Initiative Collaborative Learning***

| *Model Summary^b^* | | | | |
| --- | --- | --- | --- | --- |
| Model | R | R Square | Adjusted R Square | Std. Error of the Estimate |
| 1 | 0.397^a^ | 0.157 | 0.155 | 0.91476 |
| a. Predictors: (Constant), CCSD_Connectedness | | | | |
| b. Dependent Variable: M_LIST_InitiativeCollab | | | | |

| *ANOVA^a^* | | | | | | |
| --- | --- | --- | --- | --- | --- | --- |
| Model | | Sum of Squares | df | Mean Square | F | Sig. |
| 1 | Regression | 51.395 | 1 | 51.395 | 61.419 | <.001^b^ |
|  | Residual | 275.304 | 329 | 0.837 |  |  |
|  | Total | 326.699 | 330 |  |  |  |
| a. Dependent Variable: M_LIST_InitiativeCollab | | | | | | |
| b. Predictors: (Constant), CCSD_Connectedness | | | | | | |

| *Coefficients^a^* | | | | | | |
| --- | --- | --- | --- | --- | --- | --- |
| Model | | Unstandardized Coefficients | | Standardized Coefficients | t | Sig. |
|  |  | B | Std. Error | Beta |  |  |
| 1 | (Constant) | 1.457 | 0.229 |  | 6.366 | <.001 |
|  | CCSD_Connectedness | 0.065 | 0.008 | 0.397 | 7.837 | <.001 |
| a. Dependent Variable: M_LIST_InitiativeCollab | | | | | | |

| *Excluded Variables^a^* | | | | | | |
| --- | --- | --- | --- | --- | --- | --- |
| Model | | Beta In | t | Sig. | Partial Correlation | Collinearity Statistics |
|  |  |  |  |  |  | Tolerance |
| 1 | CCSD_Learning | -0.013^b^ | -0.208 | 0.835 | -0.012 | 0.651 |
| a. Dependent Variable: M_LIST_InitiativeCollab | | | | | | |
| b. Predictors in the Model: (Constant), CCSD_Connectedness | | | | | | |

**Table E:**

**Stepwise regression analysis: Influence of *Connectedness* and *Learning* on *Subordinate Collaborative Learning***

| *Model Summary^b^* | | | | |
| --- | --- | --- | --- | --- |
| Model | R | R Square | Adjusted R Square | Std. Error of the Estimate |
| 1 | 0.526^a^ | 0.277 | 0.275 | 0.75572 |
| a. Predictors: (Constant), CCSD_Connectedness | | | | |
| b. Dependent Variable: M_LIST_SubordCollab | | | | |

| *ANOVA^a^* | | | | | | |
| --- | --- | --- | --- | --- | --- | --- |
| Model | | Sum of Squares | df | Mean Square | F | Sig. |
| 1 | Regression | 71.947 | 1 | 71.947 | 125.976 | <.001^b^ |
|  | Residual | 187.896 | 329 | 0.571 |  |  |
|  | Total | 259.842 | 330 |  |  |  |
| a. Dependent Variable: M_LIST_SubordCollab | | | | | | |
| b. Predictors: (Constant), CCSD_Connectedness | | | | | | |

| *Coefficients^a^* | | | | | | |
| --- | --- | --- | --- | --- | --- | --- |
| Model | | Unstandardized Coefficients | | Standardized Coefficients | t | Sig. |
|  |  | B | Std. Error | Beta |  |  |
| 1 | (Constant) | 1.953 | 0.189 |  | 10.330 | <.001 |
|  | CCSD_Connectedness | 0.077 | 0.007 | 0.526 | 11.224 | <.001 |
| a. Dependent Variable: M_LIST_SubordCollab | | | | | | |

| *Excluded Variables^a^* | | | | | | |
| --- | --- | --- | --- | --- | --- | --- |
| Model | | Beta In | t | Sig. | Partial Correlation | Collinearity Statistics |
|  |  |  |  |  |  | Tolerance |
| 1 | CCSD_Learning | 0.071^b^ | 1.214 | .226 | 0.067 | 0.651 |
| a. Dependent Variable: M_LIST_SubordCollab | | | | | | |
| b. Predictors in the Model: (Constant), CCSD_Connectedness | | | | | | |

**Table F:**

**Stepwise regression analysis: Influence of CCS-D-Items on Collaborative Learning**

| *Model Summary^f^* | | | | |
| --- | --- | --- | --- | --- |
| Model | R | R Square | Adjusted R Square | Std. Error of the Estimate |
| 1 | 0.433^a^ | 0.188 | 0.185 | 0.75725 |
| 2 | 0.519^b^ | 0.269 | 0.265 | 0.71918 |
| 3 | 0.556^c^ | 0.0309 | 0.303 | 0.70055 |
| 4 | 0.571^d^ | 0.326 | 0.317 | 0.69308 |
| 5 | 0.581^e^ | 0.338 | 0.328 | 0.68789 |
| a. Predictors: (Constant), Item 15 | | | | |
| b. Predictors: (Constant), Item 15, Item 3 | | | | |
| c. Predictors: (Constant), Item 15, Item 3, Item 14 i | | | | |
| d. Predictors: (Constant), Item 15, Item 3, Item 14 i, Item 9 i | | | | |
| e. Predictors: (Constant), Item 15, Item 3, Item 14 i, Item 9 i, Item 19 | | | | |
| f. Dependent Variable: M_LIST_total_Collaboration | | | | |

| *ANOVA^a^* | | | | | | |
| --- | --- | --- | --- | --- | --- | --- |
| Model | | Sum of Squares | df | Mean Square | F | Sig. |
| 1 | Regression | 43.555 | 1 | 43.555 | 75.956 | <.001^b^ |
|  | Residual | 188.657 | 329 | 0.573 |  |  |
|  | Total | 232.211 | 330 |  |  |  |
| 2 | Regression | 62.565 | 2 | 31.282 | 60.482 | <.001^c^ |
|  | Residual | 169.647 | 328 | 0.517 |  |  |
|  | Total | 232.211 | 330 |  |  |  |
| 3 | Regression | 71.730 | 3 | 23.910 | 48.719 | <.001^d^ |
|  | Residual | 160.482 | 327 | 0.491 |  |  |
|  | Total | 232.211 | 330 |  |  |  |
| 4 | Regression | 75.614 | 4 | 18.904 | 39.353 | <.001^e^ |
|  | Residual | 156.597 | 326 | 0.480 |  |  |
|  | Total | 232.211 | 330 |  |  |  |
| 5 | Regression | 78.422 | 5 | 15.684 | 33.145 | <.001^f^ |
|  | Residual | 153.790 | 325 | .473 |  |  |
|  | Total | 232.211 | 330 |  |  |  |
| a. Dependent Variable: M_LIST_total_Collaboration | | | | | | |
| b. Predictors: (Constant), Item 15 | | | | | | |
| c. Predictors: (Constant), Item 15, Item 3 | | | | | | |
| d. Predictors: (Constant), Item 15, Item 3, Item 14 i | | | | | | |
| e. Predictors: (Constant), Item 15, Item 3, Item 14 i, Item 9 i | | | | | | |
| f. Predictors: (Constant), Item 15, Item 3, Item 14 i, Item 9 i, Item 19 | | | | | | |

| *Coefficients^a^* | | | | | | |
| --- | --- | --- | --- | --- | --- | --- |
| Model | | Unstandardized Coefficients | | Standardized Coefficients | t | Sig. |
|  |  | B | Std. Error | Beta |  |  |
| 1 | (Constant) | 2.689 | 0.108 |  | 24.913 | <.001 |
|  | Item 15 | 0.375 | 0.043 | 0.433 | 8.715 | <.001 |
| 2 | (Constant) | 2.169 | 0.134 |  | 16.235 | <.001 |
|  | Item 15 | 0.276 | 0.044 | 0.318 | 6.246 | <.001 |
|  | Item 3 | 0.261 | 0.043 | 0.308 | 6.063 | <.001 |
| 3 | (Constant) | 1.821 | 0.153 |  | 11.901 | <.001 |
|  | Item 15 | 0.261 | 0.043 | 0.301 | 6.052 | <.001 |
|  | Item 3 | 0.214 | 0.043 | 0.253 | 4.939 | <.001 |
|  | Item 14 i | 0.175 | 0.040 | 0.209 | 4.321 | <.001 |
| 4 | (Constant) | 1.618 | 0.167 |  | 9.660 | <.001 |
|  | Item 15 | 0.238 | 0.043 | 0.275 | 5.488 | <.001 |
|  | Item 3 | 0.167 | 0.046 | 0.198 | 3.656 | <.001 |
|  | Item 14 i | 0.157 | 0.041 | 0.188 | 3.885 | <.001 |
|  | Item 9 i | 0.138 | 0.049 | 0.151 | 2.844 | .005 |
| 5 | (Constant) | 1.502 | 0.173 |  | 8.689 | <.001 |
|  | Item 15 | 0.211 | 0.044 | 0.244 | 4.759 | <.001 |
|  | Item 3 | 0.130 | 0.048 | 0.154 | 2.719 | .007 |
|  | Item 14 i | 0.131 | 0.042 | 0.156 | 3.146 | .002 |
|  | Item 9 i | 0.128 | 0.049 | 0.139 | 2.631 | .009 |
|  | Item 19 | 0.138 | 0.057 | 0.139 | 2.436 | .015 |
| a. Dependent Variable: M_LIST_total_Collaboration | | | | | | |
